# Environmental control of anthraquinone–flavonoid accumulation in wild plant species: biosynthetic pathways, ecological drivers, and adaptive significance

**DOI:** 10.3389/fpls.2026.1782805

**Published:** 2026-04-01

**Authors:** Lidiia S. Samarina, Nina V. Terletskaya, Aizhan S. Mussayeva

**Affiliations:** Institute of Physiology and Genetics, Almaty, Kazakhstan

**Keywords:** anthraquinones, biosynthetic regulation, eco-metabolomics, flavonoids, phytochemical plasticity, stress adaptation, wild plant populations

## Abstract

Anthraquinones and flavonoids form an environmentally responsive, co-regulated chemical system that wild plants use to withstand multi-stress environments and structure ecological interactions. This review shows that these metabolites draw on shared precursors (especially malonyl-CoA and type III polyketide synthases) but are wired into partially distinct biosynthetic routes and regulatory networks, allowing plants to flexibly rebalance carbon flux between them under changing abiotic and biotic pressures. Across wild taxa, field and experimental data reveal that light and UV, temperature extremes, drought and flooding, edaphic heterogeneity, herbivory, pathogens, mutualists, and competitors drive predictable shifts in the anthraquinone:flavonoid ratio, generating fine-scale “phytochemical mosaics” and locally adapted chemotypes along latitudinal, altitudinal, and soil gradients. The review highlights functional complementarity rather than simple trade-offs: flavonoids predominantly buffer abiotic stress and mediate signaling, whereas anthraquinones provide high-intensity antimicrobial, antiherbivore, and allelopathic defenses, often acting through phototoxicity and soil-active residues. At the same time, pronounced intraspecific variation, strong phenotypic plasticity, and context-dependent metabolic trade-offs underscore the importance of regulatory hubs (MBW complexes, hormone cross-talk, and emerging epigenetic mechanisms) and of carbon-partitioning constraints in shaping AQ–flavonoid portfolios in nature. The review identifies major gaps—including the underrepresentation of anthraquinones in ecological genomics, a lack of multi-factor field experiments, limited integration of metabolomics with fitness and community data, and a large “dark metabolome” of uncharacterized AQ–flavonoid derivatives—and proposes an eco-metabolomic research agenda to link genes, pathways, environmental drivers, and fitness in order to predict how these dual defense systems will reorganize under rapid global change.

## 1 Introduction

Among the myriad classes of specialized metabolites in plants, anthraquinones and flavonoids stand out as two of the most significant and functionally diverse groups, combining strong redox activity, photochemical reactivity, and a wide range of targets in interacting organisms (Winkel-Shirley, 2001). While flavonoids are nearly ubiquitous across plants, anthraquinones exhibit a more mosaic distribution, often occurring in high concentrations in specific families such as the Polygonaceae, Rubiaceae, and Asphodelaceae, Fabaceae (Mao et al., 2025; Sana et al., 2025). Except the medicinal and nutritional applications, plant anthraquinones and flavanols are widely used in livestock as natural feed additives to improve animal health, enhance productivity, and reduce environmental impact (like methane production). They are considered potential alternatives to traditional antibiotics due to their antimicrobial and antioxidant properties (Onu et al., 2024).

Over the past decade, rapid advances in genomics, metabolomics, and systems biology have transformed our understanding of both anthraquinone and flavonoid metabolism in plants. High-quality reference genomes and transcriptomes for several anthraquinone-producing taxa, combined with increasingly sensitive LC–MS-based metabolomics, now allow the reconstruction of biosynthetic networks in species where these pathways were historically obscure (Kang et al., 2020; Wang et al., 2024; Chachar et al., 2024). At the same time, an expanding body of work has illuminated the complex regulatory circuits that modulate flavonoid biosynthesis in response to light, temperature, water status, nutrient availability, and pathogen attack, especially in model and crop species (Zhao et al., 2024; Shi et al., 2024). However, most modern studies treat either anthraquinones as a pharmacologically interesting but ecologically under contextualized of metabolites, or focus exclusively on flavonoids and related phenylpropanoids (Alami et al., 2024; Mao et al., 2025; Rana et al., 2025; Sana et al., 2025; Chacón-Fuentes et al., 2025). Additionally, significant portion of experimental phytochemical research is centered on agricultural crops or model species, where the primary goals are yielding optimization, quality control, and sometimes biofortification. Domestication often leads to a reduction in chemical defense diversity and stress tolerance traits (Meyer et al., 2012). In contrast, wild plants evolve under heterogeneous environmental conditions and therefore maintain broader and more plastic specialized-metabolite repertoires, as confirmed by recent comparative metabolomic studies (Dsouza et al., 2025). In wild plant communities, anthraquinones and flavonoids suggesting a potential influence on decomposition rates, nutrient cycling, and soil microbial community structure through their presence in litter and root exudates (Wang et al., 2024; Dsouza et al., 2025). Recent comparative metabolomics of wild versus cultivated medicinal plants have repeatedly shown higher chemical diversity and sometimes higher levels of bioactive compounds in wild populations, strongly suggesting that environmental complexity and genetic diversity jointly promote a richer secondary-metabolite repertoire (Wang et al., 2024; 2025; Terletskaya et al., 2024; Yerbay et al., 2025). Despite the wealth of individual studies on plant phenolics, there remains a striking lack of integrated syntheses that specifically address the environmental drivers of anthraquinone and flavonoid co-occurrence and co-regulation in wild taxa. Thus, this review seeks to address that gap by pursuing four interlinked objectives. The first objective is to evaluate how specific abiotic factors and their combinations drive anthraquinones - flavonoids branches in wild species. The second objective is to identify the regulatory “nodes”—including transcription factors, signaling pathways, hormonal crosstalk (e.g., jasmonate, abscisic acid, salicylic acid), and metabolic bottlenecks such as malonyl-CoA supply—that allow wild plants to coordinate their chemical responses to multi-factorial stress. The third objective is to distinguish, where possible, between genetically fixed traits (local adaptation) and environmentally induced, reversible responses (phenotypic plasticity) in anthraquinone and flavonoid metabolism. Building on recent advances in plant metabolomics and controlled-environment research, the review proposes an eco-metabolomic framework and experimental strategies for predicting how specialized metabolism in wild populations will shift in an era of rapid global change.

## 2 Structure, biosynthesis and regulatory architecture of anthraquinones and flavonoids in wild plants

The structural diversity of anthraquinones and flavonoids underpins their ecological efficacy and partly explains their recurrent recruitment in plant defense and signaling. Anthraquinones are characterized by a 9,10-anthracenedione skeleton, a tricyclic aromatic system that confers significant stability, planarity, and photo-activity. In wild populations, these compounds are typically stored as glycosides in vacuoles or specialized secretory structures and can be rapidly activated by β-glucosidases upon tissue damage, thereby combining safe storage with fast deployment (Zeng et al., 2024; Lu et al., 2024; Dong et al., 2024). Substitution patterns on the anthraquinone core strongly influence solubility, redox potential, and biological targets, and thus define specific ecological roles. Emodin-type anthraquinones, common in genera such as *Rumex* and *Rheum*, are characterized by substitutions at the C-1, C-3, and C-8 positions, which enhance their antimicrobial, and allelopathic properties, making them potent deterrents for vertebrate, invertebrate and competitors (Izhaki, 2002; Zeng et al., 2024; Pantovic, 2025). In contrast, alizarin-type anthraquinones, prevalent in the *Rubiaceae*, often display substitution patterns that facilitate their role as stable pigments and rhizosphere-active compounds, affecting both pollinator attraction and soil community composition (Han et al., 2001; Dong et al., 2024).

Flavonoids, conversely, are defined by a C_6_-C_3_-C_6_ backbone, consisting of two benzene rings linked by a three-carbon heterocyclic pyran ring. This basic skeleton can be extensively modified by hydroxylation, methylation, acylation, and glycosylation into several major subclasses—flavones, flavanols, flavanones, anthocyanins, isoflavones, and others—with more than 8,000 structures now catalogued in plants (Du et al., 2024). The functional versatility of flavonoids is largely attributed to this structural plasticity: subtle changes in hydroxylation pattern, glycosylation site, or acylation can modulate their capacity to scavenge reactive oxygen species (ROS), absorb ultraviolet (UV) radiation, chelate metals, or interact with proteins and membranes. For instance, ortho-dihydroxy substitution on the B-ring greatly enhances both antioxidant capacity and UV-B screening, properties that are recurrently selected in high-light or cold environments (Hurmat et al., 2025; Ninkuu et al., 2025). Importantly, both anthraquinones and flavonoids form higher-order complexes with other metabolites (e.g., tannins, terpenoids, or alkaloids) and with cell wall components, further broadening their ecological roles. In natural populations exposed to fluctuating light, temperature, moisture, and biotic pressure, their biosynthetic networks are continuously re-tuned, making anthraquinone–flavonoid metabolism an ideal model for eco-metabolomic studies (Wang et al., 2024). A critical aspect of understanding environmental control over these metabolites is the divergence—and partial convergence—in their biosynthetic origins.

### 2.1 Biosynthetic pathways and enzymatic divergence

**Figure 1:**
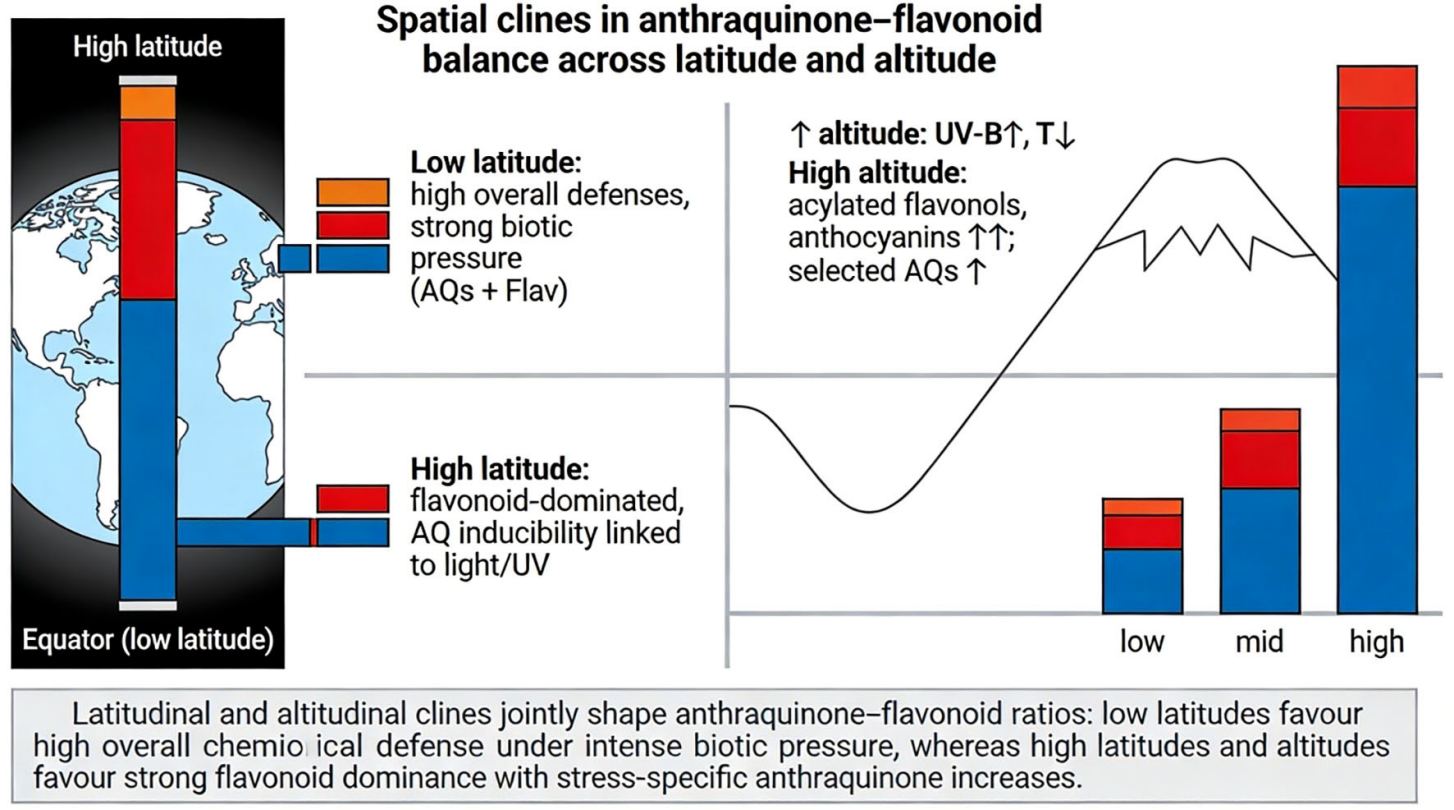
Biosynthetic architecture of AQ, anthraquinone and flavonoid pathways. This schematic summarizes the partially shared but mechanistically distinct biosynthetic organization of flavonoid and AQ, anthraquinone pathways in wild plants. Both pathways derive from primary metabolism via acetyl-CoA and the central precursor malonyl-CoA, representing a key metabolic branching node. In the flavonoid branch (blue), chalcone synthase (CHS; type III PKS) catalyzes condensation of p-coumaroyl-CoA with malonyl-CoA to form naringenin chalcone, followed by sequential hydroxylation, reduction, and tailoring reactions that generate flavonols, anthocyanins, and related subclasses. In the anthraquinone branch (red), CHS-like polyketide synthases assemble octaketide intermediates that cyclize to emodin anthrone–type scaffolds, or alternatively proceed via the shikimate/OSB pathway in certain taxa. Subsequent oxidation and modification steps produce structurally diverse AQs. Despite sharing malonyl-CoA and type III PKS ancestry, the two branches diverge in chain length, ring formation chemistry, and ecological specialization.

The metabolic origin of plant anthraquinones is bifurcated into two primary routes: the polyketide pathway and the shikimate/o-succinylbenzoate (OSB) pathway (Han et al., 2001) (**Figure 1**). The polyketide pathway, prevalent in Polygonaceae (e.g., *Rheum*, *Rumex*), Fabaceae (e.g., *Senna*), and several Asphodelaceae, utilizes a type III polyketide synthase (PKS) to catalyze the iterative condensation of one acetyl-CoA starter with typically seven malonyl-CoA extender units (Lian et al., 2025). This reaction leads to an octaketide intermediate that undergoes enzyme-guided folding and cyclization to form the anthracene nucleus, followed by tailoring steps such as oxidation, O-methylation, and glycosylation that generate the enormous structural diversity of plant anthraquinones (Abe and Morita, 2010; Wang et al., 2024).

Conversely, in Rubiaceae (e.g., *Rubia cordifolia*), the A- and B-rings of AQs are derived from chorismic acid and α-ketoglutarate via the OSB intermediate, while the C-ring is assembled from isopentenyl diphosphate (IPP) via the MEP pathway (Zhang et al., 2022). Recent full-length transcriptome sequencing of *R. cordifolia* identified hundreds of candidate genes spanning the shikimate, TCA, MVA/MEP, and downstream tailoring pathways, confirming that multiple primary-metabolic modules are recruited into AQ biosynthesis and that expression of these modules differs markedly between above- and below-ground organs (Lian et al., 2025). Multi-omics work in *Reynoutria japonica* and other knotweeds similarly points to dispersed but co-regulated gene sets rather than a single compact biosynthetic gene cluster, suggesting that AQ pathways in plants often emerge from distributed networks rather than classical microbial-style clusters (Chen et al., 2025). This functional divergence allows the plant to sequester defense-related AQs in reproductive tissues—where protection of fitness-critical organs is paramount—while maintaining UV-protective flavonoids in vegetative organs.

Flavonoids, in contrast, are synthesized exclusively via the phenylpropanoid pathway, which branches from aromatic amino acid metabolism when phenylalanine ammonia-lyase (PAL) converts L-phenylalanine to *trans*-cinnamic acid. A series of hydroxylation and activation steps yields p-coumaroyl-CoA, which then enters the committed step of flavonoid biosynthesis: condensation with three molecules of malonyl-CoA catalyzed by chalcone synthase (CHS), a specialized type III PKS, to form naringenin chalcone (Winkel-Shirley, 2001). Subsequent reactions mediated by chalcone isomerase, flavanone 3-hydroxylase, flavonol synthase, dihydroflavonol 4-reductase, and numerous decorating enzymes diversify the flavonoid pool into flavones, flavonols, anthocyanins, and other subclasses, each with distinct ecological roles.

The malonyl-CoA node is conceptually central for crosstalk between type III PKS-derived pathways, including flavonoids and anthraquinones, yet direct quantitative validation of malonyl-CoA partitioning in planta remains limited. In microbial and heterologous systems, increasing malonyl-CoA supply is a well-known lever that alters flavonoid yields, demonstrating that malonyl-CoA availability can act as a measurable bottleneck (Zha et al., 2009). Thus, from an eco-evolutionary perspective, these biosynthetic architectures show that anthraquinones and flavonoids share both enzymes (type III PKS) and precursors (malonyl-CoA) yet have evolved partially independent upstream modules (shikimate/OSB versus phenylpropanoid) in different species (Wang et al., 2024). How these hybrid networks are wired and environmentally regulated in wild populations remains a key question.

### 2.2 Regulatory networks: the MBW complex and beyond

**Figure 2:**
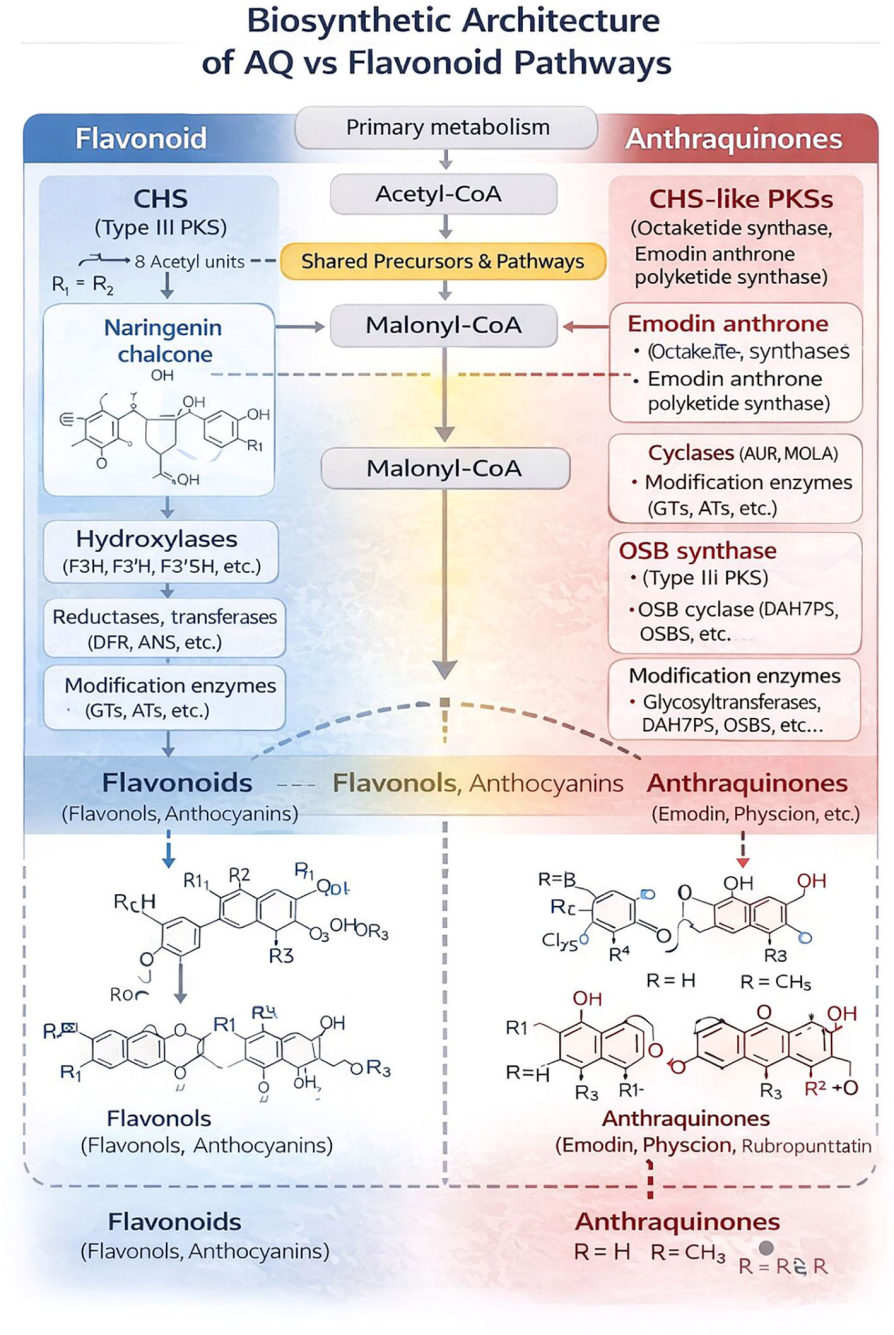
Regulatory hubs integrating environmental signals: flavonoid *vs* anthraquinone branches. This schematic illustrates the hierarchical integration of environmental cues into flavonoid and AQ, anthraquinone biosynthesis. External drivers—including light, UV-B, drought, and biotic stress—converge on a hormonal signaling layer composed of JA, jasmonic acid , SA, salicylic acid; ABA, abscisic acid, Ca^2+^ signaling, and ROS, reactive oxygen species. These signals interact with an epigenetic layer involving chromatin remodeling (e.g., histone acetylation), DNA methylation, and regulatory RNAs (e.g., miRNAs), which modulate transcriptional accessibility and pathway responsiveness. Downstream, TF, transcription factor hubs—such as the MBW complex, MYB, bZIP, and NPR1—coordinate branch-specific activation of biosynthetic genes. The flavonoid branch (blue) is typically associated with UV protection, antioxidant buffering, and light-responsive regulation, whereas the anthraquinone branch (red) is more strongly linked to pathogen defense and stress-inducible responses. The diagram emphasizes regulatory convergence, cross-talk between layers, and the modular control architecture governing metabolic allocation.

The transcriptional regulation of flavonoids is governed by the highly conserved MYB–bHLH–WD40 (MBW) complex, consisting of R2R3-MYB transcription factors, bHLH (basic helix–loop–helix) proteins, and WD40 repeat proteins (**Figure 2**). This ternary complex binds to the promoters of structural genes such as *CHS*, *CHI* (chalcone isomerase), *F3H* (flavanone 3-hydroxylase), and *DFR* (dihydroflavanol 4-reductase), thereby conferring tight spatial and temporal control over flavonoid accumulation in different tissues, developmental stages, and environmental contexts (Hichri et al., 2011; Du et al., 2024; Ninkuu et al., 2025). In *Senna tora*, genome-wide analysis has identified a massive expansion of the type III PKS family through tandem duplication, resulting in over 30 PKS genes (Kang et al., 2020). While some of these genes encode canonical CHS enzymes responsible for flavonoid production in leaves, others (e.g., CHS-L2/3) are highly expressed in seeds and exhibit modified active-site cavities capable of accommodating longer polyketide chains required for anthraquinone biosynthesis (Kang et al., 2020). Recent integrative studies have expanded this view, showing that MBW complexes form part of larger regulatory modules that include upstream signaling kinases, hormone-responsive transcription factors, and chromatin modifiers, especially under drought, cold, and nutrient stress (Mao et al., 2025; Wang et al., 2025).

Importantly, not all MYB family members act as activators: R3- and specific R2R3-MYB transcriptional repressors lacking activation domains can compete with activator MYBs for interaction with bHLH and WD40 partners, thereby dampening flavonoid pathway activity or redirecting flux toward particular branches (e.g., flavanols versus anthocyanins). This dynamic balance between activator and repressor MYBs enables plants to fine-tune flavonoid profiles in response to subtle environmental cues—a feature likely to be especially important in wild populations exposed to complex, temporally variable stresses (Bulgakov et al., 2024). Emerging work also implicates epigenetic mechanisms in flavonoid regulation: DNA methylation and histone modifications at key phenylpropanoid loci can be modulated by stress, shaping both acute responses and transgenerational “stress memory” in wild and semi-wild species (Fan et al., 2024).

In the case of anthraquinones, the regulatory architecture is less well characterized, but is increasingly linked to similar transcription factor families and signaling pathways (Wang et al., 2024). While genome-enabled studies have identified core biosynthetic genes (e.g., CHS-like polyketide synthases) and strong co-expression modules, direct demonstration of specific transcription factors binding and activating anthraquinone biosynthetic promoters is still scarce (Kang et al., 2020). Recent transcriptome-driven work in *Rheum* species has nominated candidate R2R3-MYB and bZIP transcription factors correlated with anthraquinone accumulation, but these studies primarily provide association and expression-based evidence rather than direct TF–promoter validation (Zhao et al., 2024; Tang et al., 2024). In *Rheum palmatum*, comparative transcriptomics and genome data have identified *RpMYB* factors that are significantly upregulated during methyl-jasmonate (MeJA)-induced AQ accumulation, and functional assays suggest that these MYBs directly activate key polyketide and tailoring genes (Zhao et al., 2024). Moreover, calcium-dependent signaling components such as Ib-rolB/C in *Rubia cordifolia* callus cultures have been shown to upregulate AQ biosynthesis, linking classical defense signaling pathways to anthraquinone production in a manner reminiscent of flavonoid induction. In *Ophiorrhiza pumila*, the transcription factor OpMYB1 co-regulates the biosynthesis of seco-iridoids and anthraquinones, suggesting the existence of “master switch” regulators that integrate multiple stress-responsive pathways into coordinated metabolic programs (Rohani et al., 2016). A recent study on *Rheum officinale* infected by smut disease revealed that the global regulator RoNPR1 (Nonexpressor of Pathogenesis-Related genes 1) interacts with bZIP transcription factors (RoTGA1/2) to activate both flavonoid and anthraquinone biosynthesis (Wang et al., 2025). This coordinated response ensures a multi-layered chemical defense, where flavonoids quench infection-induced ROS and reinforce cell walls, while anthraquinones act as direct antimicrobial agents targeting membrane integrity or respiratory enzymes. Such dual-pathway activation under natural pathogen attack underscores the importance of regulatory crosstalk for understanding AQ–flavonoid dynamics in wild populations.

Consequently, at present there are few convincing examples of the same MYB/bHLH factors directly co-regulating both anthraquinone and flavonoid biosynthetic genes. Regulatory overlap therefore likely occurs more frequently at higher levels (shared hormonal/stress signaling and chromatin state) than through fully shared terminal transcriptional regulators. Given the limited evidence for shared terminal transcription factors across both pathways, hormone-mediated cross-talk likely represents a more plausible mechanism for coordinated responses. Jasmonate signaling is a well-established upstream driver of flavonoid/anthocyanin regulation through JAZ-centered repression modules that interface with MBW-controlled genes, while simultaneously modulating chromatin states at key regulators (Hichri et al., 2011; Liao et al., 2022; Huang et al., 2026). For anthraquinones, jasmonate responsiveness is frequently reported at the transcript level for biosynthetic genes in medicinal taxa, yet promoter binding and regulatory circuit logic remain incompletely resolved (Kang et al., 2020; Zhao et al., 2024). Thus, at present, hormone cross-talk can be treated as a realistic upstream “hub” for co-activation, whereas direct co-regulation by identical MYB/bHLH factors remains largely hypothesis-generating.

Epigenetic mechanisms offer an additional layer by which pathway co-expression could be coordinated. For flavonoids/anthocyanins, multiple studies demonstrate that DNA methylation and chromatin-modifying enzymes modulate expression of key transcriptional regulators and pathway genes, thereby influencing pigment and flavonoid output (Telias et al., 2011; Liao et al., 2022). However, epigenetic coordination between anthraquinone and flavonoid pathways remains largely untested, representing a major priority for future integrative studies. Taken together, these studies indicate that anthraquinone and flavonoid pathways are embedded in shared regulatory networks that respond to hormones (JA, SA, ABA), Ca^2+^ signals, redox status, and environmental cues. Yet the extent to which the same regulators control both pathways—and how this control varies among wild taxa adapted to different environments—remains poorly resolved.

### 2.3 Metabolic, ecological, and adaptive trade-offs in anthraquinone–flavonoid allocation

The concept of a “trade-off” between anthraquinones (AQs) and flavonoids is frequently invoked in plant secondary metabolism, yet it is often applied implicitly and without explicit distinction among underlying mechanisms. In AQ–flavonoid systems, trade-offs may arise at multiple, partially independent levels, including metabolic constraints, ecological cost–benefit dynamics, and adaptive allocation shaped by natural selection. Disentangling these levels is essential for identifying scenarios in which competition between the two pathways is real and measurable, as opposed to cases where apparent trade-offs reflect functional complementarity.

Metabolic trade-off. Flavonoid biosynthesis requires three malonyl-CoA units per molecule, whereas AQ biosynthesis typically requires seven, rendering AQ production substantially more carbon- and energy-intensive (Winkel-Shirley, 2001; Abe and Morita, 2010). This difference establishes a clear mechanistic basis for metabolic competition under conditions of limited carbon flux. Experimental studies demonstrate that malonyl-CoA availability can constrain the simultaneous upregulation of multiple polyketide pathways. Acetyl-CoA carboxylase (ACC), the rate-limiting enzyme in malonyl-CoA biosynthesis, represents a key metabolic bottleneck, and manipulation of ACC activity alters carbon allocation between primary metabolism, growth, and secondary metabolite production (Zha et al., 2009). In *Senna tora*, genome-enabled analyses revealed tissue-specific expansion and expression of CHS-like polyketide synthases associated with anthraquinone biosynthesis, accompanied by reduced expression of canonical chalcone synthase genes in seeds, consistent with a developmental reallocation of malonyl-CoA toward AQ production at the expense of flavonoids (Kang et al., 2020). This represents true metabolic trade-offs, grounded in precursor limitation and enzymatic competition rather than purely regulatory coincidence.

Ecological trade-off. Anthraquinones often provide strong antimicrobial, anti-herbivore, and allelopathic effects, but their synthesis, storage, and detoxification impose measurable energetic and physiological costs. High constitutive investment in carbon-intensive defenses is frequently associated with reduced growth rates or delayed reproduction under low-stress conditions, consistent with classical growth–defense trade-off theory (Herms and Mattson, 1992). In contrast, flavonoids often serve multifunctional roles, including UV screening, antioxidant buffering, and signaling, allowing them to enhance plant performance even in the absence of direct biotic antagonists (Agati et al., 2012). This functional asymmetry results in ecological trade-offs in allocation strategies: in environments dominated by chronic pathogen pressure or intense below-ground competition, AQ investment may yield higher defensive benefits despite higher costs, whereas in habitats characterized by high irradiance or oxidative stress, flavonoid-dominated strategies may provide greater net fitness returns. Many plants mitigate these ecological trade-offs through spatial partitioning, preferentially allocating AQs to roots, rhizomes, or reproductive tissues while maintaining flavonoid-rich profiles in photosynthetic organs (Han et al., 2001).

Adaptive trade-off. Along environmental gradients, populations often exhibit reciprocal investment in AQ- versus flavonoid-dominated chemotypes, suggesting that selection favors different chemical strategies depending on local abiotic and biotic pressures (Zidorn, 2010; Pellissier et al., 2013). Importantly, many wild species resolve adaptive trade-offs through phenotypic plasticity rather than fixed specialization. Inducible anthraquinone production in response to herbivory or pathogen attack, combined with constitutive or light-responsive flavonoid accumulation, allows plants to minimize continuous metabolic costs while retaining defensive capacity when needed (Agrawal, 2007; Moreira et al., 2025). Such plasticity blurs the boundary between trade-off and complementarity, emphasizing that AQ–flavonoid allocation operates along a continuum shaped by environmental context, developmental stage, and resource availability.

Taken together, available evidence indicates that trade-offs between anthraquinones and flavonoids are context-dependent and hierarchical (**Table 1**). Metabolic trade-offs are most evident under precursor limitation; ecological trade-offs arise from differential costs and benefits of defense strategies; and adaptive trade-offs reflect evolutionary optimization across heterogeneous environments. Explicitly distinguishing among these levels clarifies why AQ and flavonoid pathways may compete under certain conditions yet appear complementary at the whole-plant scale.

**Table 1:** Comparative biosynthetic and regulatory features of flavonoids and anthraquinones.

Feature	Flavonoids	Anthraquinones
Core pathway	Phenylpropanoid	Polyketide or OSB/shikimate
Key enzyme	Chalcone synthase (CHS)	Type III PKS (CHS-like)
Malonyl-CoA demand	3 units	~7 units
Main regulators	MYB–bHLH–WD40 (MBW) complex	MYB, bZIP, JA/SA-responsive TFs (less characterized)
Dominant functions	Photoprotection, signaling, antioxidant activity	Antimicrobial, anti-herbivore, allelopathic defense
Spatial distribution	Epidermis, vacuole, vascular tissues	Roots, rhizomes, secretory structures

### 2.4 Scientific gaps and future directions

- Enzymatic validation in non-model taxa. While thousands of “CHS-like” and PKS genes have been identified through SMRT and genome sequencing in species such as *Rubia cordifolia* and *Reynoutria japonica*, only a small subset has been biochemically characterized for anthraquinone formation *in vitro* (Chen et al., 2025; Lian et al., 2025). Establishing substrate specificities, product profiles, and kinetic parameters for these enzymes—especially under physiologically relevant conditions—remains a major task.
- Regulatory crosstalk between pathways. It remains unclear whether specific “repressor” MYBs (e.g., MYB4-like factors) or other transcriptional regulators can selectively downregulate flavonoid or anthraquinone branches to favor the other during particular stress scenarios (Wang et al., 2025). Integrated transcriptomic and promoter-binding studies in wild or semi-wild conditions are needed to determine whether shared regulators orchestrate complementary or antagonistic responses of the two pathways.
- Spatiotemporal dynamics in the field. The influence of circadian rhythms, seasonality, and realistic multi-stress combinations (e.g., UV + drought + herbivory) on the AQ–flavonoid ratio is almost entirely undocumented in natural populations (Rai et al., 2025; Hao et al., 2025; Mao et al., 2025). Longitudinal field sampling combined with high-throughput metabolomics and time-resolved transcriptomics would greatly advance this area.
- Epigenetic control and stress memory. The role of DNA methylation, histone modification, and small RNAs in modulating AQ and flavonoid pathways in wild species is only beginning to be explored (Bulgakov et al., 2024). Whether similar mechanisms underlie anthraquinone induction and whether stress-induced epigenetic states contribute to transgenerational “priming” of specialized metabolism in wild taxa are open questions.
- Integration with primary metabolism and fitness. Finally, there is a lack of studies linking detailed biosynthetic models to whole-plant performance and fitness in the field (Chacón-Fuentes et al., 2025; Wang et al., 2024).

## 3 Abiotic drivers of anthraquinone and flavonoid accumulation

### 3.1 Light intensity and spectral quality: the role of UV radiation

Light is perhaps the most critical environmental regulator of specialized metabolism because it simultaneously serves as an energy source and a stressor. New experimental studies using controlled light spectra and excess-light treatments in wild and semi-wild species show similar parallel increases in flavonoids and other photoreactive metabolites, reinforcing the view that light quality and quantity jointly sculpt AQ–flavonoid profiles (Qu et al., 2025; Hurmat et al., 2025).

For flavonoids, the relationship with light is well established through the activation of photoreceptors such as UVR8 (Ultraviolet-B Resistance 8), cryptochromes, and phytochromes, which collectively perceive UV-B, blue, and red/far-red wavelengths (Agati et al., 2012). UV-B radiation ( 280–315 nm) triggers a signaling cascade in which UVR8 monomers interact with COP1 and downstream transcription factors, inducing the expression of *CHS* (chalcone synthase), *FLS* (flavanol synthase), and other phenylpropanoid genes, leading to the accumulation of dihydroxy-substituted flavanols like quercetin and kaempferol derivatives (Pollastri and Tattini, 2011).

While flavonoids act primarily as shields and modulators, AQs often exhibit photodynamic properties. In many wild taxa, AQs are sequestered in glandular trichomes, resin ducts, or specialized idioblasts. Upon exposure to high light, these compounds can become photo-excited and generate ROS locally, which may confer phototoxic effects against pathogens or herbivores (Izhaki, 2002). This spatial compartmentation allows plants to weaponize sunlight at tissue interfaces while limiting damage to internal tissues, and may be particularly important in open, high-UV habitats where surface-associated attackers are common (Nazif et al., 2000; Wang et al., 2024). For example, in wild populations of *Polygonum aviculare* the concentration of emodin-type AQs correlates positively with light intensity, suggesting a coordinated upregulation of polyketide and phenylpropanoid pathways to maximize both photoprotection and chemical defense (Zidorn, 2010).

### 3.2 Temperature extremes and altitudinal gradients

Altitude is a complex environmental variable integrating decreasing temperature with increasing UV-B radiation, wind exposure, and often reduced atmospheric pressure. Wild plants along altitudinal transects frequently demonstrate a “chemical elevation effect,” with systematic changes in their flavonoid and anthraquinone profiles as elevation increases. Low temperatures often lead to an “energy overflow” where carbon fixed during brief favorable periods cannot be fully utilized for growth, thereby being redirected into the phenylpropanoid pathway and other carbon-rich secondary metabolites (Jaakola and Hohtola, 2010). Anthocyanins, in particular, are accumulated in high-altitude leaves, providing a thermal advantage by increasing leaf absorptance of solar radiation and by scavenging ROS generated during cold-induced photoinhibition (Agati et al., 2021; Yu et al., 2021). On the other hand, heat stress exerts almost opposite pressures on specialized metabolism. High temperatures can destabilize complex flavonoids and accelerate their enzymatic and non-enzymatic degradation, often resulting in a shift toward smaller, more stable phenolic acids and certain AQs. In *Hypericum* species, for example, heat stress has been shown to paradoxically increase the concentration of hypericin (an AQ derivative) under certain regimes, which may help stabilize membranes and modulate ROS production, although sustained heat generally depresses anthocyanin levels (Skyba et al., 2012).

Recent integrative analyses suggest that temperature effects on flavonoid and AQ accumulation are strongly context-dependent, influenced by concomitant light intensity, water status, and developmental stage (Gaude and Jalmi, 2025; Zhan et al., 2022). Wild rhubarb (*Rheum aristatum*) growing at altitudes exceeding 4,000 m produces a distinctive profile of acylated flavanols and glycosylated AQs that is largely absent in lowland congeners. Research indicates that the high concentrations of physcion and chrysophanol in these alpine populations are adaptive responses to combined stress from sub-zero night temperatures, intense daytime UV radiation, and nutrient-poor soils (Kumar et al., 2026). Similar altitudinal shifts in phenylpropanoid and anthraquinone profiles have been reported in other high-mountain medicinal plants, highlighting altitude as a natural “experiment” in multi-stress modulation of specialized metabolism (Chen et al., 2024).

### 3.3 Water availability: drought and flooding dynamics

Water deficit is a major driver of metabolic shifts and a central determinant of anthraquinone and flavonoid accumulation in wild plants. Under drought, plants close their stomata, reducing $CO_{2}$ assimilation and generating an internal excess of excitation energy in the photosystems. To prevent the resulting oxidative burst, plants upregulate flavonoid biosynthesis, deploying these compounds as “mobile antioxidants” and redox buffers in leaves, stems, and roots (Nakabayashi et al., 2014). Numerous studies now document drought-induced increases in flavonols, flavones, and anthocyanins in both model and wild species, often correlated with enhanced drought tolerance (Vicente and Boscaiu, 2018).

The drought response is mediated in large part by the phytohormone abscisic acid (ABA), which activates bZIP transcription factors such as AREB/ABF that cross-talk with MBW complexes and other regulators to boost flavonoid production (Aslam et al., 2022). In parallel, JA and ethylene signaling can promote or fine-tune phenylpropanoid pathway activation, and emerging evidence indicates that small RNAs and long non-coding RNAs modulate anthocyanin and broader flavonoid biosynthetic genes under drought, cold, and salinity stress (Moroldo et al., 2024; Gaude and Jalmi, 2025). Moderate drought can increase AQ and flavonoids concentrations, especially in leaves and roots, whereas severe, prolonged desiccation leads to a metabolic collapse and reduction in specialized-metabolite titers (Radha and Laxmipriya, 2014; Samarina et al., 2023). This suggests a non-linear relationship where mild to moderate stress acts as an elicitor, but stress beyond a threshold compromises the biosynthetic capacity of the plant which is consistent with our recent studies on stress responses of tea plant (Shkhalakhova et al., 2026; Kuzmina et al., 2025; Samarina et al., 2024).

Flooding and hypoxia remain comparatively understudied in the context of AQ and flavonoid metabolism, despite many AQ-rich taxa inhabiting riparian or periodically waterlogged habitats. Available data for phenylpropanoids suggest that waterlogging can both downregulate and reconfigure flavonoid profiles, depending on species and stress duration, and that ROS and ethylene signaling play central roles which was displayed several crops (Gong et al., 2022). In Fabaceae, wild xerophyte Senna tora, combined methyl jasmonate treatment and wounding strongly induced flavonoid-pathway transcripts and metabolites, whereas several major anthraquinones remained comparatively stable over the same time window, highlighting stimulus-specific and potentially non-parallel regulation of the two branches (Chang et al., 2024). Additionally, in *Senna tora*, AQs act not only as antioxidants but also as signaling molecules that modulate the root microbiome and recruit beneficial mycorrhizal fungi under drought stress (Kang et al., 2020). Multi-omics studies in other drought-tolerant medicinal plants support the idea that root-localized phenylpropanoids and polyketides help structure drought-adapted microbiomes (Kumar et al., 2024; Chen et al., 2024). Translating these insights to AQ–flavonoid dynamics in wild *Polygonaceae* or *Rheum* species is a promising but largely unexplored area.

### 3.4 Soil factors: nutrients, pH, salinity, and heavy metals

Soil properties impose strong and often fine-scaled constraints on plant metabolism. The “carbon–nutrient balance hypothesis” (CNBH) proposes that when nitrogen (N) or phosphorus (P) is limiting, plants redirect excess carbon into secondary metabolites, particularly carbon-rich, nitrogen-poor phenolics (Bryant et al., 1983). Consistent with this hypothesis, low N availability typically results in increased accumulation of carbon-based secondary metabolites (CBSMs) such as flavonoids and AQs, as plants divert carbon away from nitrogen-intensive protein synthesis (Koricheva et al., 1998). Recent work further indicates that specific N and P regimes can modulate not just total phenolic content but also the relative proportions of different flavonoid subclasses and possibly AQ chemotypes in wild and cultivated medicinal plants (Villamarin-Raad et al., 2023).

Salinity stress, driven by high concentrations of soluble salts in the root zone, induces both ionic and osmotic stress. Flavonoids and AQs help stabilize cell membranes, chelate toxic ions, and act as osmoprotectants and antioxidants under saline conditions. In *Rubia cordifolia*, salinity has been shown to specifically induce the OSB pathway and increase the production of alizarin-type AQs, suggesting that salt stress can serve as a targeted elicitor of AQ biosynthesis. Moderate salinity stress drastically increased the activities of all antioxidant enzymes and contents of antioxidant molecules including total phenols, and flavonoids in medicinal plant *Moringa oleifera* (Azeem et al., 2023). Similar salt-induced increases in anthraquinone accumulation have been observed in *Cassia* suspension cultures, where NaCl treatment elevated AQ levels while reducing growth, highlighting the trade-off between biomass and secondary-metabolite output under ionic stress (Nazif et al., 2000).

Soil pH and redox conditions also influence AQ and flavonoid dynamics by altering metal availability, microbial activity, and the stability of exuded metabolites (Moroldo et al., 2024). Species such as *Rumex acetosella* growing on metalliferous soils rich in Zn, Cu, or Cd exhibit high levels of flavonoids in their root exudates and tissues. These compounds can chelate metal ions in the rhizosphere, limit their uptake, or mitigate oxidative damage induced by metal-catalyzed ROS formation. Furthermore, AQs like emodin possess metal-binding sites and have been shown to form complexes with transition metals, suggesting they may play a similar, yet underappreciated, role in edaphic specialization and metal tolerance (Raghuveer et al., 2023). Collectively, these findings indicate that soil-related factors interact with light and water regimes to define local environmental niches, within which AQ–flavonoid portfolios are tuned in a highly context-dependent manner.

### 3.5 Scientific gaps in abiotic-stress research

- Multi-factorial interference. Most experiments still manipulate a single variable (e.g., UV-B alone), whereas in natural settings UV-B is often accompanied by high temperature, drought, and nutrient stress. The interactive (synergistic, additive, or antagonistic) effects of combined stresses on AQ–flavonoid ratios are poorly resolved (Chen et al., 2024; Gaude and Jalmi, 2025).
- Understudied stresses: flooding and hypoxia. While drought is extensively studied, the effects of waterlogging, hypoxia, and fluctuating water tables on anthraquinone and flavonoid metabolism remain almost entirely unknown, despite many *Polygonaceae* and *Rheum* species inhabiting riparian or wetland zones. Addressing this gap will be essential for predicting responses of AQ-rich flora to altered flooding regimes under climate change.
- Spectral specificity and canopy structure. There is limited information on how changes in the red:far-red (R:FR) ratio in dense canopies affect AQ synthesis and the balance among flavonoid subclasses, even though shade-avoidance signaling via phytochromes is known to reshape phenylpropanoid pathways. Understanding spectral effects is crucial for interpreting metabolic shifts in forest-dwelling wild species and in transition zones between open and shaded habitats (Hurmat et al., 2025).
- Epigenetic memory and transgenerational effects. Evidence is only beginning to accumulate that abiotic stress experienced by parent plants leads to “primed” flavonoid profiles in offspring through DNA methylation, histone modifications, and small RNA-mediated mechanisms (Bulgakov et al., 2024). Whether similar epigenetic mechanisms modulate anthraquinone production, and how stable such stress memories are in wild populations across generations, remains largely unexplored.
- Linking metabolites to fitness under field conditions. Finally, there is a shortage of studies that directly relate environment-induced shifts in AQ–flavonoid profiles to fitness components such as survival, growth, reproduction, and competitive ability *in situ*. Combining field manipulations, long-term demographic monitoring, and high-resolution metabolomics will be crucial to move from descriptive patterns to mechanistic, predictive models of how abiotic factors shape specialized metabolism in wild plant communities (**Table 2**).

**Table 2:** Typical responses of flavonoids and anthraquinones to major abiotic drivers in wild plants.

Abiotic driver	Typical response of flavonoids	Typical response of anthraquinones	Ecological interpretation
High light/UV radiation	Strong induction via UVR8–COP1 signaling; accumulation of flavonols (e.g., quercetin derivatives) that act as UV screens and antioxidants	Often increased in surface tissues or secretory structures; photoreactive AQs may enhance phototoxic defense against pathogens or herbivores	Complementary roles: flavonoids protect plant tissues, AQs contribute to defensive phototoxicity
Low temperature/high altitude	Increased flavonols and anthocyanins; improved ROS scavenging and photoprotection under cold-induced photoinhibition	Certain glycosylated AQs accumulate in alpine populations, potentially contributing to oxidative stress tolerance and antimicrobial defense	Cold environments favor carbon-rich secondary metabolism
Heat stress	Often decreased anthocyanins due to thermal instability; shift toward smaller phenolics	In some taxa AQ derivatives increase and may stabilize membranes or modulate ROS	Heat can shift balance from phenylpropanoids toward polyketide-derived metabolites
Drought	Frequently increased flavonols and flavones; ABA-mediated activation of phenylpropanoid pathway	Moderate drought may stimulate AQ accumulation, especially in roots; severe drought reduces both pathways	Flavonoids buffer oxidative stress; AQs may participate in root defense and microbiome interactions
Salinity/ionic stress	Increased antioxidant flavonoids and phenolics; stabilization of membranes and ROS detoxification	In some species (e.g., Rubia), salinity induces AQ biosynthesis via OSB pathway	Both metabolite classes contribute to oxidative stress tolerance
Low nutrient availability (N or P)	Increased carbon-based phenolics consistent with the carbon–nutrient balance hypothesis	AQ accumulation may also increase due to carbon surplus and reduced growth	Secondary metabolism acts as a carbon sink
Heavy metals	Chelation of metals and mitigation of ROS via flavonoids	Certain AQs may bind metals and contribute to detoxification in metalliferous soils	Chemical defense intersects with metal tolerance mechanisms

## 4 Biotic interactions: the chemical theatre of defense and symbiosis

In the complex ecological networks of wild ecosystems, anthraquinones (AQs) and flavonoids act as primary mediators of inter-organismal communication and defense. Unlike abiotic stressors, which are often pervasive and predictable (e.g., seasonal UV-B cycles), biotic pressures are characterized by high specificity, rapid evolutionary “arms races,” and multi-trophic complexity involving herbivores, pathogens, mutualists, and competitors. Anthraquinones are notoriously toxic or deterrent to generalist herbivores, often causing intestinal distress, reduced growth, or mortality in insects and vertebrate grazers (Izhaki, 2002). Flavonoids, although often less acutely toxic, modulate herbivore behavior and physiology by inhibiting detoxification enzymes, acting as feeding deterrents, or mimicking hormone signals, and also contribute to resistance against pathogens as phytoalexins synthesized *de novo* upon infection. In addition, flavonoids mediate complex community interactions: they contribute to floral pigmentation and scent, shaping pollinator visitation networks, and they function as root-exuded signals that recruit beneficial soil microbes, including nitrogen-fixing bacteria and mycorrhizal fungi (Hassan and Mathesius, 2012; Tang et al., 2024; Chacón-Fuentes et al., 2025).

In wild populations, the accumulation of these metabolites is sharply up-regulated upon perception of herbivore-associated molecular patterns (HAMPs) or microbe-associated molecular patterns (MAMPs), and is orchestrated via jasmonate (JA), salicylic acid (SA), and ethylene signaling networks (Fürstenberg-Hägg et al., 2013). Recent advances highlight that AQs and flavonoids rarely act alone; instead, plants deploy complex cocktails whose composition changes over time and space, generating a dynamic “chemical landscape” that shapes biotic interactions at multiple trophic levels (Speed, Ruxton et al., 2025; Jacoby et al., 2021; Patil et al., 2024; Kumar et al., 2024).

### 4.1 Chemical defense against herbivory

Anthraquinones, particularly emodin-type compounds, are notorious for their purgative effects on vertebrate and invertebrate digestive systems. Emodin stimulates chloride secretion in the gut, leading to rapid water loss and diarrhea, which acts as a potent post-ingestive deterrent (Izhaki, 2002). In insects, AQs can interfere with mitochondrial electron transport and redox cycling, generating superoxide radicals within the midgut, leading to oxidative damage, reduced feeding, and mortality (Malik and Müller, 2016). Structure–activity studies show that hydroxylation patterns and glycosylation strongly influence toxicity, suggesting that wild plants can fine-tune AQ deterrence through relatively small structural modifications (Qun et al., 2023; Khaliq et al., 2023; Wang et al., 2024).

Flavonoids play critical roles in modulating herbivore growth and behavior. Isoflavonoids, for instance, can act as endocrine disruptors in mammalian herbivores due to their structural similarity to estrogen (e.g., genistein), altering reproductive and metabolic physiology (Dixon and Steele, 1999). In insect herbivores, quercetin and kaempferol can inhibit cytochrome P450 monooxygenases and glutathione-S-transferases, the very enzymes relied upon for detoxifying plant secondary metabolites (Schuler et al., 2011). Recent work further indicates that flavonoids can function as feeding deterrents or antifeedants at ecologically realistic concentrations (Vicente and Boscaiu, 2018; Kumar et al., 2024; Patil et al., 2024).

For example, the wild dock (*Rumex oblongifolius*) co-occurs with the specialist leaf beetle *Gastrophysa viridula*. Studies show that while the beetle has evolved mechanisms to sequester or excrete emodin, high concentrations of AQs in leaves significantly reduce the growth rate and survival of generalist larvae, illustrating a trade-off between specialist tolerance and generalist resistance (Costan et al., 2023). When the plant is attacked, it exhibits systemic induction of both AQs and flavonoids in younger, more valuable leaves, consistent with the “Optimal Defense Theory,” which predicts preferential allocation of costly defenses to tissues with highest fitness value. Such inducible, tissue-specific responses highlight the importance of dynamic regulation rather than static constitutive levels in wild systems (Khaliq et al., 2023).

### 4.2 Pathogen resistance: antimicrobial and antifungal efficacy

Wild plants are under constant assault from fungi, bacteria, viruses, and oomycetes. The rigid aromatic structures of AQs and the redox-active, often amphipathic nature of flavonoids provide a multi-layered chemical barrier to infection (Pang et al., 2021). Many anthraquinones exhibit direct antimicrobial activity by intercalating into microbial DNA, inhibiting topoisomerases, or disrupting cell membrane integrity. Structure–activity studies show that hydroxylation and halogenation patterns strongly modify antibacterial and antifungal potency, with certain aloe-emodin and rhein derivatives demonstrating broad-spectrum activity against Gram-positive and Gram-negative bacteria. Particularly, in species such as *Rubia cordifolia*, alizarin-type AQs inhibit fungal biofilm formation and spore germination, thereby preventing colonization of root tissues and reducing infection severity (Qun et al., 2023; Chandrasekhar et al., 2023; Lian et al., 2025Additionally, in wild populations of *Senna tora*, the concentration of the anthraquinone physcion in seeds is inversely correlated with the success of soil-borne fungal pathogens. Physcion inhibits succinate dehydrogenase in the mitochondrial respiratory chain of fungi, effectively “starving” the pathogen of energy during critical infection phases (Kang et al., 2020). This seed-localized AQ defense is particularly relevant in wild habitats where seed persistence in soil and exposure to a diverse seed-pathogen community strongly influence recruitment.

Flavonoids often function as phytoalexins—compounds synthesized *de novo* in response to pathogen attack. For example, the isoflavonoid medicarpin is rapidly accumulated in legumes where it inhibits the germination and hyphal growth of fungal pathogens (Ahuja et al., 2010). Flavonoids can also modulate plant immune signaling by affecting SA and JA pathways and by influencing ROS dynamics at infection sites, thereby integrating local and systemic acquired resistance. Recent work shows that flavonoid catabolism is also important: differential breakdown of flavonoids can generate smaller phenolic acids with direct antimicrobial properties or act as signals that fine-tune defense responses (Chandrasekhar et al., 2023; Rana et al., 2025).

### 4.3 Mutualistic interactions: signaling and the microbiome

Not all biotic interactions are antagonistic; specialized metabolites are equally essential for recruiting and managing beneficial partners that enhance plant fitness. Recent work shows that diverse microbes—including endophytes and rhizobacteria—can enhance secondary-metabolite accumulation by modulating hormone levels, nutrient uptake, and expression of biosynthetic genes, suggesting that AQ–flavonoid profiles in wild plants are emergent properties of plant–microbe networks rather than plant genomes alone (Lv et al., 2024; Rana et al., 2025). Flavonoids and, increasingly, AQs are recognized as key components of below-ground and above-ground signaling webs (Pang et al., 2021). Flavonoids are classic signaling molecules in the recruitment of nitrogen-fixing rhizobia. Secreted flavonoids bind to the rhizobial NodD protein, triggering expression of nodulation (nod) genes and the synthesis of Nod factors, which in turn initiate nodule formation on legume roots (Hassan and Mathesius, 2012). Beyond this canonical role, flavonoids in root exudates act as chemoattractants, carbon sources, and selective inhibitors that shape the broader rhizosphere microbiome, favoring beneficial microbes while suppressing potential pathogens.

Several anthraquinones, including emodin, chrysophanol, and physcion, are known to be released from roots or decaying below-ground tissues and exhibit strong antimicrobial or phytotoxic activity in laboratory and pot-based assays (Inderjit et al., 2021; Izhaki, 2002). These properties are consistent with potential roles in suppressing soil-borne pathogens or mediating plant–plant allelopathic interactions via root exudates. However, direct field-based evidence linking root-exuded AQs to shifts in rhizosphere microbial community composition or function remains scarce.

Arbuscular mycorrhizal fungi (AMF) can significantly alter the phytochemical profile of their host plants. This “bio-induction” often results in higher levels of both flavonoids and anthraquinones, as AMF improve nutrient (especially phosphorus) acquisition and activate host defense pathways. In *Aloe vera*, for example, inoculation with AMF increases the concentration of aloin (an AQ) by up to ~30%, likely reflecting both improved P nutrition and JA/SA-linked defense signaling (Radha and Laxmipriya, 2014). In Fabaceae, *Senna tora* shows stimulus-dependent decoupling between branches: jasmonate- and wounding-related signaling preferentially amplifies flavonoid responses, while anthraquinone accumulation can be less responsive on the same timescale—consistent with a division between rapid redox/photoprotective buffering (flavonoids) and more context- or tissue-specific deployment of polyketide-derived defenses (Chang et al., 2024). However, in contrast to the well-established roles of flavonoids in symbiotic interactions, the below-ground ecological functions of anthraquinones (AQs) remain comparatively underexplored.

### 4.4 Community dynamics and allelopathy

In wild communities, plants use their chemical arsenal to compete for space, light, and nutrients, and to influence successional trajectories. This “chemical warfare” or allelopathy can dictate species composition in natural habitats and contribute to invasion success (Khaliq et al., 2023). Certain flavones and other flavonoids released into the soil can chelate essential nutrients, alter auxin transport, or disrupt cell-cycle progression in neighboring plants, effectively suppressing their growth (Buer et al., 2010). Because flavonoid exudation is highly plastic and responsive to environmental cues, wild plants may dynamically adjust allelopathic pressure according to competition intensity, nutrient status, or herbivory (Patil et al., 2024; Pantovic, 2025).

Considering AQs, the emodin and other AQs release into the soil through leaf litter, root exudates, or rhizome decay. They inhibit germination, root elongation, and seedling establishment of neighboring species by interfering with mitochondrial respiration, disrupting membrane integrity, and altering hormone signaling (Izhaki, 2002). Reviews of *Rumex* spp. allelopathy underscore that AQ-rich residues reduce growth of neighboring crops and weeds, highlighting their potential role in natural weed suppression and, conversely, in competitive dominance of certain wild Rumex populations (Khaliq et al., 2023). The invasive wild plant *Polygonum cuspidatum* dominates ecosystems partly due to its high AQ content. Emodin and other AQs released into the soil create chemically hostile “zones of exclusion” where native species experience reduced germination and growth, potentially facilitating monoculture formation (Inderjit et al., 2021). In Fabaceae, *Senna tora* shows stimulus-dependent decoupling between branches: jasmonate- and wounding-related signaling preferentially amplifies flavonoid responses, while anthraquinone accumulation can be less responsive on the same timescale. These examples suggest that anthraquinone–flavonoid systems may play a role in shaping community structure, invasion dynamics, and ecosystem processes (Chen et al., 2025).

### 4.5 Scientific gaps in biotic-interaction research

- Synergistic and antagonistic effects. There is limited knowledge on whether flavonoids enhance or mitigate the bioavailability, stability, or toxicity of AQs against herbivores and pathogens, or vice versa (Moreira et al., 2025). Applying mixture-toxicity frameworks and network pharmacology to natural metabolite blends would help resolve these interaction effects (Pang et al., 2021).
- The “third trophic level.” Very few studies examine how plant AQs and flavonoids affect predators and parasitoids of herbivores, such as ladybirds or parasitic wasps. Multi-trophic experiments that track metabolite transfer along food chains are needed to clarify these indirect effects.
- Field induction kinetics and natural variability. The real-world kinetics of how AQ–flavonoid levels shift during natural multi-day herbivore outbreaks, pathogen epidemics, or community succession events in wild populations is largely unknown (Agrawal, 2007). High-frequency field sampling combined with targeted and untargeted metabolomics would allow quantification of these dynamic responses (Speed and Ruxton 2025).
- Linking chemistry to fitness and community outcomes. Finally, there is a shortage of studies that connect AQ–flavonoid variation explicitly to plant fitness (survival, fecundity) and community-level metrics (diversity, invasion resistance) under natural biotic regimes. Integrating chemical ecology with demographic and community-ecology approaches will be essential to understand how these metabolites shape—and are shaped by—the biotic context of wild ecosystems (Jacoby et al., 2021; Pang et al., 2021).
- Deficiency of AQs studies. While laboratory assays demonstrate that many AQs possess antimicrobial and allelopathic activity, few studies have directly quantified their release into the rhizosphere or measured their effects on microbial community structure, pathogen suppression, or plant–plant interactions under natural conditions. Addressing this gap will require integrative field experiments that combine root exudate profiling, soil microbiome analysis, and manipulative removal or addition of specific AQ compounds.

## 5 Intraspecific variation and local adaptation in specialized metabolism

The survival of wild plant species across heterogeneous landscapes depends on their ability to match their chemical phenotypes to local environmental pressures. Intraspecific variation—the diversity in trait values among individuals or populations of the same species—is the raw material upon which natural selection acts and is particularly pronounced for specialized metabolites that mediate interactions with climate, soils, herbivores, pathogens, and mutualists (Yerbay et al., 2025). For AQs and flavonoids, this variation manifests as complex spatial clines and mosaics, driven by a tension between genetic differentiation (local adaptation) and phenotypic plasticity (the ability of a single genotype to produce different phenotypes in response to environmental cues) (Nicotra et al., 2010). Recent metabolomic surveys show that even neighboring populations can differ strikingly in their AQ–flavonoid fingerprints, underscoring the importance of fine-scale environmental and genetic factors (Jangpangi et al., 2025; Dixon and Dickinson, 2024; Skubel et al., 2020).

### 5.1 Patterns of spatial variation: latitudinal and altitudinal clines

Geographic gradients in light, temperature, moisture, and herbivory exert consistent selective pressures that often result in predictable clines in secondary-metabolite concentrations (Sun, Fernie 2024). The Latitudinal Gradient in Herbivory (LGH) hypothesis suggests that plants at lower latitudes (tropics) face more intense and specialized herbivory, leading to higher levels of chemical defenses compared with temperate populations (Moles et al., 2011). This pattern is robust for several alkaloids and tannins, but research into AQ–flavonoid systems reveal more nuanced outcomes. Particularly, in wild populations of *Hypericum perforatum*, latitudinal variation in hypericin (an AQ derivative) is more strongly correlated with day length and UV-B intensity than with herbivore pressure alone; northern populations exhibit lower baseline hypericin levels but higher inducibility, consistent with a strategy of inducible, light-linked defense in shorter growing seasons (Seyis et al., 2020). Similar studies in other medicinal species suggest that climatic drivers (light, temperature) and biotic drivers (herbivory, pathogens) jointly shape latitudinal chemotype distributions rather than acting independently (Jangpangi et al., 2025; Sun, Fernie 2024).

**Figure 3:**
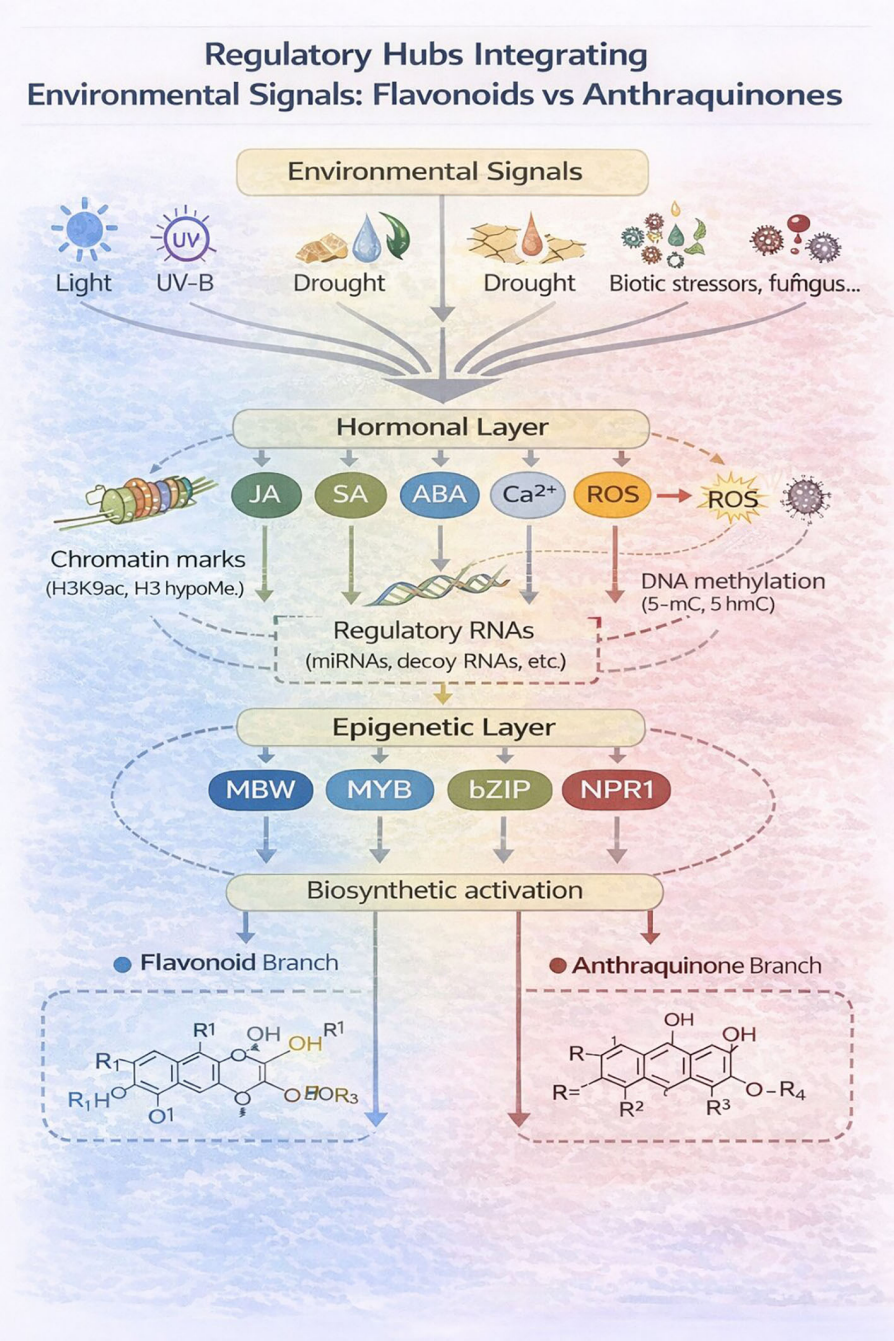
Spatial clines in the anthraquinone–flavonoid balance across latitude and altitude in wild plants. The figure presents a qualitative synthesis of how latitudinal and altitudinal gradients influence the relative investment in anthraquinones (AQs, red segments) and flavonoids (Flav, blue segments) in wild species. The coloured bar segments indicate the relative contribution of each metabolite class (no quantitative values are implied).

Altitudinal gradients provide condensed versions of latitudinal clines, where temperature drops and UV-B radiation increases over short distances (**Figure 3**). In Polygonaceae, wild populations of *Rumex* species show distinctive “metabolic shifts” at higher elevations. Populations above ~2,500 m consistently produce higher ratios of glycosylated flavanols (e.g., quercetin-3-O-glucoside) to aglycones, interpreted as a dual adaptation: glycosylation increases solubility and vacuolar storage capacity, improving UV screening, while the enhanced antioxidant potential mitigates cold-induced oxidative stress (Zidorn, 2010; Pellissier et al., 2013). Comparable altitudinal patterns have been documented for anthraquinone derivatives in *Rheum* and *Rubia* species, where alpine populations accumulate higher levels of particular AQs than lowland conspecifics, likely reflecting combined selection by UV-B, low temperature, and short growing seasons (Alami et al., 2024; Jangpangi et al., 2025).

### 5.2 Phenotypic plasticity and adaptive bet-hedging

In environments where stress regimes are unpredictable—such as erratic rainfall, episodic frost events, or sporadic insect outbreaks—plants may evolve phenotypic plasticity rather than fixed “high-defense” phenotypes. Plasticity allows plants to defer investment in expensive AQs until reliable cues indicate elevated risk. In *Rheum* species, induction of emodin and related AQs in response to mechanical wounding or herbivore attack is highly plastic. Populations from relatively stable, low-disturbance environments tend to show low plasticity and higher constitutive defense, whereas populations from frequently disturbed habitats exhibit strong inducibility, allowing them to conserve resources when herbivores are absent but mount robust defenses when attacked (Agrawal and Weber, 2015). Similar patterns have been reported for flavonoid and anthocyanin induction across environmental gradients, suggesting that reaction norms of specialized metabolism are themselves targets of selection (Koski et al., 2024).

In extremely fluctuating or unpredictable environments, some wild species employ “bet-hedging,” where a single population produces individuals with varying chemical profiles regardless of immediate cues (Sultan, 2000). This strategy ensures that at least some individuals are pre-equipped for “worst-case” scenarios such as severe pathogen outbreaks or extreme drought. This is particularly evident in seed banks of *Senna tora*, where seeds from the same mother plant show substantial variation in AQ-rich seed coats, resulting in staggered germination timing and differential resistance to soil fungi (Kang et al., 2020). Such intra-family metabolic heterogeneity can increase long-term lineage persistence in stochastic environments, albeit at the cost of producing some mismatched phenotypes each generation.

### 5.3 The role of local adaptation in “chemical races”

The concept of “chemical races” or chemotypes is central to wild-plant ecology. Within a single species, distinct populations may evolve entirely different chemical solutions to broadly similar ecological problems (Dixon and Dickinson, 2024). For example, wild *Polygonum aviculare* exhibits distinct chemotypes along coastal–inland transects. Coastal populations subjected to high salinity, wind-borne salt spray, and intense irradiance have evolved flavonoid-heavy profiles dominated by methylated flavones and highly glycosylated flavanols, which are more effective at stabilizing membranes, mitigating ion toxicity, and providing UV screening under osmotic stress. In contrast, inland populations of the same species prioritize production of AQs and associated phenolics for defense against soil-borne pathogens and root herbivores prevalent in humid forest soils (Zidorn, 2010). This divergence represents local adaptation driven primarily by edaphic and microclimatic selection pressures, and illustrates how different branches of the same metabolic network (AQs *vs*. flavonoids) can be differentially favored in neighboring environments.

Comparable chemitypic differentiation has been reported in other medicinal and invasive species, where populations differ in suites of flavonoids, AQs, or other phenolics along climate or soil gradients. These “chemical races” can have with potential implications for community interactions and ecosystem processes, herbivore specialization, and efficacy of wild germplasm as a resource for pharmacology (Skubel et al., 2020; Jangpangi et al., 2025).

Although intraspecific variation in anthraquinone and flavonoid profiles is increasingly documented, the genetic basis of such chemotypic differentiation remains unevenly resolved. For flavonoid pathways, natural variation in metabolite composition has been linked to polymorphisms in both structural genes (e.g., chalcone synthase and flavonoid hydroxylases) and regulatory loci, particularly R2R3-MYB transcription factors, with allele-specific effects on pathway flux and metabolite ratios demonstrated in several plant systems (Hichri et al., 2011). In contrast, for anthraquinones, evidence for genotype–metabolite associations remains limited. Genome-enabled studies in anthraquinone-producing taxa have identified expanded polyketide synthase (PKS) gene families and strong co-expression modules, yet direct links between specific SNPs or allelic variants in PKS or regulatory genes and ecotype-specific anthraquinone profiles are largely lacking (Kang et al., 2020; Zhao et al., 2024). As a result, the extent to which AQ–flavonoid ratios reflect heritable genetic differentiation versus environmentally induced plasticity remains unresolved in most systems.

Beyond standing genetic variation, transgenerational plasticity provides an additional mechanism through which AQ–flavonoid systems may respond to environmental heterogeneity. In several plant species, parental exposure to abiotic or biotic stress has been shown to alter secondary-metabolite accumulation and stress responsiveness in offspring, often mediated by epigenetic modifications such as DNA methylation and histone marks (Lämke and Bäurle, 2017; Kambona et al., 2023). These effects have been most extensively documented for phenolic compounds, including flavonoids, where stress-induced metabolic priming can persist across one or more generations. Comparable evidence for transgenerational regulation of anthraquinone biosynthesis is currently lacking. Nevertheless, given the shared reliance of AQ and flavonoid pathways on chromatin-sensitive regulatory networks, parental effects represent a plausible yet largely unexplored contributor to intraspecific variation and evolutionary potential in AQ-producing taxa. Together, genetically encoded chemotypic variation and transgenerational plasticity define the evolutionary capacity of AQ–flavonoid systems. Disentangling these mechanisms will be essential for predicting whether observed metabolic variation represents short-term physiological flexibility or a substrate for adaptive divergence under persistent environmental change.

### 5.4 Scientific gaps and future research directions

- Genomic basis of variation. Although chemical clines and chemotypes are increasingly documented, the specific alleles and loci (e.g., SNPs in *CHS*, PKS genes, regulatory MYBs, or transporters) underlying this variation remain largely unknown in wild taxa (Koski et al., 2024; Wager and Li, 2018). Extending such approaches to AQ-producing wild species would greatly advance understanding of the genetic architecture of specialized-metabolite clines.
- Epigenetic inheritance and transgenerational plasticity. Multi-generational studies are scarce, making it difficult to assess whether “transgenerational plasticity”—where parental exposure to stress alters offspring AQ or flavonoid levels—is a significant component of local adaptation. Recent work in flavonoid-rich species demonstrates that DNA methylation and histone modifications at key phenylpropanoid genes can differ between populations and are associated with both SNP variation and stress history, influencing flavonoid accumulation (Jump and Peñuelas, 2005; Yu et al., 2021). Whether similar epigenetic mechanisms modulate anthraquinone pathways, and how stable these marks are under changing climates, remains a major open question (Bulgakov et al., 2024; Yu et al., 2021).
- Non-linear and multidimensional responses. Many studies still search for simple linear clines (e.g., “more altitude = more flavonoid”), yet environmental interactions are often threshold-based, non-linear, and multidimensional. Secondary-metabolite responses to combined drivers such as temperature, moisture, CO_2_, and ozone frequently show non-additive patterns, complicating inference from single-gradient analyses (Sun and Fernie 2024). Future work should use response-surface modelling and multivariate statistics to capture how multiple variables interact to shape “metabolic landscapes” across space.
- Sampling biases and undersampled biomes. Most detailed work on intraspecific variation still comes from temperate species in Europe and North America, or from a handful of model and crop systems. Tropical wild species, which inhabit more complex and diverse biotic environments and often display extreme chemical diversity, remain a “black box” in terms of their AQ–flavonoid adaptive strategies (Moreira et al., 2025). Expanding sampling to underrepresented regions and populations is essential for a more complete and globally relevant view.
- Linking chemistry, genotype, and fitness. Finally, relatively few studies directly connect metabolomic variation with genotypes and fitness outcomes (survival, fecundity, competitive ability) in natural settings (Ruan et al., 2025; Hao et al., 2025). Integrative frameworks combining population genomics, mGWAS, transcriptomics, and field-based demographic monitoring offer a promising route to bridge this gap and to predict how AQ–flavonoid diversity will respond to ongoing climate change.

## 6 Discussion

### 6.1 Trade-offs versus functional complementarity in AQ–flavonoid systems

A recurrent pattern highlighted throughout this review is the frequent co-occurrence of anthraquinones (AQs) and flavonoids within the same species, populations, and even tissues, despite the potential for competition between these pathways (Winkel-Shirley, 2001; Wang et al., 2024; Sana et al., 2025). This observation raises a central interpretative question: how can evidence for metabolic and ecological trade-offs (Section 2.3) be reconciled with the apparent functional complementarity of AQ–flavonoid portfolios observed in wild plants? One key factor is biological scale. Trade-offs are most clearly detected at the level of metabolic constraints or short-term resource limitation, whereas field-based metabolomic patterns integrate responses across tissues, developmental stages, and fluctuating environments (Herms and Mattson, 1992; Moreira et al., 2025). As a result, competition for shared resources such as malonyl-CoA may not manifest as simple negative correlations between AQ and flavonoid concentrations at the whole-plant or population level, a pattern repeatedly reported in comparative studies of wild taxa (Zidorn, 2010; Wang et al., 2024).

Spatial and temporal partitioning further moderates apparent trade-offs. Numerous studies indicate that AQs and flavonoids are differentially allocated across organs and phenological stages, with AQs enriched in roots, rhizomes, seeds, or reproductive tissues, while flavonoids dominate in photosynthetically active organs exposed to abiotic stress (Han et al., 2001; Kang et al., 2020). Such partitioning is consistent with strategies that reduce direct metabolic conflict while preserving complementary defensive and physiological functions.

Phenotypic plasticity adds another layer of complexity. Inducible AQ accumulation in response to herbivory or pathogen attack, combined with constitutive or light-responsive flavonoid production, suggests that trade-offs may be transient and context-dependent rather than fixed properties of a given genotype (Agrawal, 2007; Agati et al., 2012; Yerbay et al., 2025). Consequently, static metabolomic snapshots may underestimate the extent to which AQ–flavonoid allocation shifts dynamically in response to environmental cues, a limitation increasingly emphasized in recent eco-metabolomic studies (Sun and Fernie, 2024; Rai et al., 2025).

From an ecological perspective, the simultaneous presence of AQs and flavonoids is consistent with functional complementarity rather than redundancy. Flavonoids predominantly mitigate abiotic stress through UV screening, antioxidant buffering, and signaling functions, whereas AQs often provide high-intensity antimicrobial, anti-herbivore, and allelopathic defenses (Agati et al., 2012; Izhaki, 2002; Khaliq et al., 2023). The co-expression of both classes may therefore broaden the spectrum of stress tolerance and defense across heterogeneous environments, even when their biosynthesis draws on overlapping metabolic resources (Moreira et al., 2025; Sana et al., 2025). A predictive eco-metabolomic framework for AQ–flavonoid systems should combine (i) multifactorial experimental designs that manipulate at least two interacting abiotic drivers, (ii) high-resolution metabolomic profiling across tissues and developmental stages, and (iii) integrative analytical approaches linking metabolite data to physiological performance and fitness proxies.

In summary, anthraquinone and flavonoid pathways in wild plants appear to constitute a flexible, context-dependent chemical system. Trade-offs between them are real but conditional, operating most clearly at specific metabolic or ecological scales, while functional complementarity often dominates at higher levels of biological organization. Explicit recognition of this scale dependence is essential for interpreting AQ–flavonoid variation in nature and for avoiding oversimplified conclusions about metabolic competition in complex ecological settings.

### 6.2 The “multi-stress synergy” model

Classical experimental designs often adopt a “one stress–one metabolite” framework, yet wild plants rarely experience isolated stressors. The synthesis across Sections 2 and 3 instead points to a multi-stress synergy, where combinations of stressors (e.g., high UV-B + drought, heat + salinity, herbivory + pathogen attack) elicit metabolic outputs that are non-additive and often qualitatively distinct from single-stress responses (Zhan et al., 2022; Sana et al., 2025; Sun, Fernie 2024).

Mechanistically, several layers of evidence support this model:

- Hormonal cross-talk: Drought-induced ABA signaling primes the phenylpropanoid pathway, enhancing flavonoid responsiveness to subsequent UV-B exposure, while JA and SA pathways coordinate herbivore and pathogen-induced activation of both flavonoid and AQ branches (Agati et al., 2012; Aslam et al., 2022).
- Structural adaptation: In alpine environments, simultaneous cold and high radiation select for acylated flavonoids, which exhibit enhanced stability, membrane association, and antioxidant capacity relative to non-acylated analogues (Pellissier et al., 2013).
- Regulatory hubs: MBW complexes, NPR1/NPR-like regulators, and other transcriptional “hubs” identified in Section 1 integrate multiple upstream signals into coordinated regulation of phenylpropanoid and polyketide genes, providing a plausible mechanistic basis for multi-stress integration (Wang et al., 2025).

Under this multi-stress synergy paradigm, local adaptation is understood not as optimization to a single dominant stressor, but as the evolution of regulatory architectures that can produce appropriate AQ–flavonoid combinations under characteristic multi-factor regimes. To move beyond descriptive inference, response-surface and factorial experimental designs provide a powerful framework for quantifying how secondary-metabolite allocation responds to interacting environmental drivers. Such approaches allow detection of non-linearities, trade-offs, and threshold effects that are not apparent in single-factor studies. Response-surface models have already been successfully applied to predict plant physiological and metabolic responses to combined stresses, including drought × heat and nutrient × CO_2_ interactions (Zandalinas et al., 2021). Extending these designs to AQ–flavonoid systems would enable explicit forecasting of metabolite reorganization across realistic climate-change scenarios rather than *post hoc* interpretation of isolated drivers.

### 6.3 Phytochemical plasticity

Anthropogenic climate change is reshaping the environmental context in which AQ–flavonoid systems evolved. Rapid shifts in temperature, precipitation, CO_2_, and disturbance regimes threaten to decouple historical cues from current selective environments, with uncertain consequences for phytochemical plasticity (Sun, Fernie 2024). Climate change is expected to expose plants to novel combinations of stressors, such as elevated temperature, episodic drought, and increased atmospheric CO_2_. These factors can exert opposing effects on carbon availability, oxidative stress, and defense investment, suggesting that AQ–flavonoid trade-offs may shift in non-intuitive ways under future conditions. Rising temperatures may disrupt the tight coupling between light and temperature that historically shaped flavonoid induction. If flavonoids remain primarily light-induced but are thermally destabilized or downregulated under chronic heat stress, photoprotective capacity could decline even as UV loads remain high, especially in mid-latitude and alpine regions (Lemanowicz et al., 2026). Conversely, elevated temperatures may enhance the production or persistence of certain AQs, potentially shifting the defensive balance toward more biotically oriented chemistry. Predictive eco-metabolomic models that integrate multi-factor stress responses with carbon allocation and defense function are therefore essential for anticipating how chemical defense portfolios will reorganize in natural and managed ecosystems (Krasensky and Jonak, 2012; Zandalinas et al., 2021). Increased atmospheric CO_2_ is expected to raise tissue C:N ratios, which under the Carbon–Nutrient Balance (CNB) hypothesis should favor accumulation of carbon-rich secondary metabolites such as AQs and flavonoids (Bryant et al., 1983; Koricheva et al., 1998). However, recent experiments show mixed outcomes: in some cases, elevated CO_2_ increases total phenolic content, whereas in others rapid biomass accumulation leads to “metabolic dilution,” where per-mass concentrations of defensive phenolics decline despite higher total plant pools, with potentially increased vulnerability to herbivores and pathogens (Agrawal and Weber, 2015). This suggests that predictions based solely on CNB are insufficient and must be refined to include growth rate, tissue turnover, and multi-stress interactions. As climate niches shift, locally adapted chemotypes may experience maladaptation or demographic decline, especially in long-lived wild perennials with limited dispersal. Species whose AQ–flavonoid profiles are tightly tuned to narrow altitude or microclimate bands may be particularly vulnerable, risking loss of unique chemical diversity before it is characterized. This underscores the urgency of documenting chemotypic variation and its environmental drivers across species ranges while intact (Jangpangi et al., 2025).

### 6.4 Key research gaps and future priorities

The apparent dominance of Polygonaceae, Rubiaceae, and Asphodelaceae in the present review reflects a pronounced taxonomic bias in the underlying literature rather than an assumption of their unique functional importance. These families have historically served as model systems for anthraquinone research due to their high and constitutive AQ accumulation, well-characterized chemical diversity, and frequent use in medicinal and ecological studies (Izhaki, 2002; Han et al., 2001; Kang et al., 2020). As a result, they provide the most detailed insights into AQ–flavonoid co-occurrence and regulation currently available. In contrast, several anthraquinone-producing families such as Asteraceae are comparatively underrepresented in integrative studies of AQ–flavonoid dynamics. In Asteraceae, anthraquinones are typically embedded within complex secondary-metabolite matrices dominated by sesquiterpene lactones and flavonoids, complicating attribution of ecological function to individual compound classes (Cheng and Cheng, 2015; Zidorn, 2010). Reports of anthraquinones (e.g., emodin-type compounds in some Asteraceae are presented however, integrative datasets that jointly quantify AQ and flavonoid rebalancing across environmental gradients in these taxa remain scarce compared with Polygonaceae/Rubiaceae/Fabaceae. This uneven taxonomic coverage has important evolutionary implications. Families with high constitutive anthraquinone investment may represent lineages in which the dual AQ–flavonoid system has become canalized, whereas families with sporadic or tissue-specific AQ occurrence may rely more strongly on inducible or context-dependent deployment of these metabolites. Expanding comparative analyses beyond current model families will therefore be essential for resolving whether AQ–flavonoid complementarity represents a conserved evolutionary strategy or a set of lineage-specific solutions shaped by distinct ecological and phylogenetic constraints.
